# Anisomycin has a potential toxicity of promoting cuproptosis in human ovarian cancer stem cells by attenuating YY1/lipoic acid pathway activation

**DOI:** 10.7150/jca.77445

**Published:** 2022-10-24

**Authors:** Xiaoli Nie, Haiyang Chen, Ying Xiong, Juan Chen, Te Liu

**Affiliations:** 1 Shanghai Geriatric Institute of Chinese Medicine, Shanghai University of Traditional Chinese Medicine, Shanghai 200031, China.; 2 Department of Obstetrics and Gynecology, Xinhua Hospital Affiliated to Shanghai Jiao Tong University School of Medicine, Shanghai 200092, China.; 3 Gongli Hospital Affiliated to the Second Military Medicical University in Pudong New Area of Shanghai City, Shanghai 200135, China.

**Keywords:** Ovarian cancer stem cells, anisomycin, Yinyang 1(YY1), lipoic acid pathway, cuproptosis

## Abstract

Ovarian cancer is a highly malignant gynecologic tumor that seriously endangers women's health. We previously demonstrated that anisomycin significantly inhibited the activity of ovarian cancer stem cells (OCSCs) in vitro and in vivo. In the present study, anisomycin treatment of OCSCs significantly reduced ATP and T-GSH content; and increased pyruvate, LPO, and MDA. Anisomycin also significantly inhibited the proliferation of OCSCs in vitro, and its effect was similar to that of elesclomol and buthionine sulfoximine (BSO), suggesting that it has the potential to promote cuproptosis of OCSCs. Our subsequent cDNA microarray analysis results showed that anisomycin significantly reduced the transcriptional levels of genes that protect copper metabolism and cuproptosis, including the PDH complex, metallothionein, lipoid acid pathway, and FeS cluster proteins. Bioinformatics analysis revealed that four core factors (lipoic acid pathway FDX1, DLD, DLAT, PDH), and transcription factor YY1 were highly expressed in ovarian cancer tissues and were significantly correlated with an unfavorable prognosis. Further analysis depicted multiple YY1-recognized motif basic sites as existing in the promoters of the above four factors. In addition, the expression levels of YY1 in the tissue samples from ovarian cancer patients were significantly positively correlated with the expression levels of FDX1, DLD, DLAT, PDHB, and other genes. Finally, the analysis of the peripheral blood exosome database disclosed that the contents of the four key factors of YY1 and the lipoic acid pathway in the peripheral blood exosomes of patients with ovarian cancer were significantly elevated relative to those of normal healthy individuals. Therefore, our molecular biology experiments combined with bioinformatics analysis results suggest that the direct target of anisomycin-induced cuproptosis in ovarian cancer stem cells is probably a YY1 transcription factor. By inhibiting the expression and activity of YY1, anisomycin could not activate the transcriptional activity of the core genes of the lipoic acid pathway (i.e.,FDX1, DLD, DLAT, and PDHB), and induced the accumulation of cytotoxic substances, eventually leading to potential cuproptosis in ovarian cancer stem cells.

## Introduction

Ovarian cancer is a highly malignant gynecologic tumor that seriously affects women's health 1, 2. In recent years the incidence of ovarian cancer has increased in young female, with commensurately greater reproductive injury 1, 2. Many investigators have reported a group of cellular subsets similar to embryonic stem cells in ovarian cancer tissue samples that markedly express markers such as CD44, CD133, and c-Kit (CD117); and that possess stem-cell “stemness.” However, these cells also portray the characteristics of highly proliferative, invasive, and tumorigenic capabilities inherent totumor cells, such that they are referred to as ovarian cancer tumor stem cells (OCSCs) 3-5. Because of the great heterogeneity of OCSCs and their robust tolerance to traditional chemotherapeutic agents, it is particularly important to develop efficient targeted drugs to kill OCSCs. Cell biologists conventionally indicate that cell death can be divided into numerous types. According to different triggering mechanisms, cell death can be divided into apoptosis, necrosis, programmed cell death, pyroptosis, and autophagy 6, 7. Many factors trigger cell death, including inflammatory factors, cytotoxins, environmental radiation, and chemical toxins 8-10. Recent studies have shown that metal ions also induce specific cell death, such as iron-induced cell death or ferroptosis 11-13; and cell death caused by the accumulation of copper ions is designated copper-induced cell death or cuproptosis 14-17. Copper is a basic element that is necessary for the biological processes of life. In mammals, copper binds to enzymes to assist in blood clotting, hormonal maturation, and the cellular processing of energy; and also participates in many biological behaviors 16. However, excessive copper ions in the body can damage cells and induce pathological changes 18, 19. Compared with normal resting cells, cancer cells show more vigorous cell divisions and demonstrate a higher demand for copper. Studies have shown elevated levels of copper in tumors or serum in animal models and patients with multiple cancers, including gynecologic, genitourinary, and digestive cancers 14-17. Copper-chelating agents are therefore expected to be developed as adjuvant drugs for the treatment of cancer.

Anisomycin (3,4-Pyrrolidinediol, 2- [(4-methoxyphenyl) methyl] -, 3-acetate, (2R, 3S, 4S) is an antibiotic purified from *Streptomyces griseus* 20 that inhibits peptide bond formation and inhibits protein synthesis by binding to 60S ribosomal subunits 20-22. Anisomycin is also a c-Jun N-terminal kinase (JNK) activator that enhances the phosphorylation of JNK 23, 24. Anisomycin exerts significant cytotoxic effects on eukaryotes and some protozoa 20, and several studies have indicated that anisomycin not only promotes the production of amyloid β (Aβ42) but also inhibits the proliferation and invasion of various tumor cells 20, 21, 25-27. Yu et al. found that anisomycin inhibited the proliferation of Jurkat T cells by stimulating the expression of p53, p21, and p27 signals and by blocking the entry of signal-initiated cells into S and G2/M phases 28; and Seo et al. reported that anisomycin augmented apoptosis of renal tumor cells by down-regulating Bcl-2, c-FLIP, and Mcl-1 26. Liu et al. also demonstrated that anisomycin induced apoptosis in glucocorticoid-resistant acute lymphoblastic leukemia cells via phosphorylation of mitogen-activated protein kinase p38 and JNK 24, 29, and these authors revealed that anisomycin induced glioma cell death by downregulating the PP2A catalytic subunit in vitro 29. Our previous studies showed that anisomycin augmented intracellular Aβ42 concentrations by stimulating the expression of lncRNA BACE1-AS1, thereby inhibiting proliferation and invasion by OCSCs 21. We further reported that anisomycin inhibited the activation of downstream components of the Notch1 pathway by attenuating the molecular sponge effect of the lncRNA-Meg3/miR-421/PDGFRA axis, ultimately inhibiting angiogenesis, proliferation, and invasion by ovarian cancer cells 27. Thus, anisomycin is a potential chemotherapeutic agent.

According to the aforementioned results, we implemented molecular biological methods to screen the expression of genesclosely related to cuproptosis using a gene expression profile chip, and combined this with a large-sample database of tumor patients and bioinformatics analysis methods. This allowed us to analyze in detail the potential of anisomycin in inducing cuproptosis in ovarian cancer stem cells by activating the specific transcription factor YY1 and transactivating important genes related to the lipoic acid pathway. We also evaluated the expression levels of cuproptosis-related genes in exosomes from peripheral blood between patients with ovarian cancer and healthy individuals, providing potential diagnostic markers for the early screening of ovarian cancer.

## Material and Methods

### Isolation and culture of primary OCSCs

We conducted this experiment according to previously described methods 4, 27. Briefly, surgically isolated tissues from fourovarian cancer patients were minced, digested with 0.25% trypsin (Gibco, Gaithersburg, MD, USA), and centrifuged at 1500 r/min for 5min. The cell precipitates were collected and incubated with mouse anti-human CD133-FITC antibodies and rabbit anti-human CD44-APC antibodies (e-Bioscience Inc., San Diego, CA, USA) in vitro at 4°C for 30min. We then sorted the CD44+/CD133+ OCSCs from the sample by flow cytometric analysis.

### Treatment of OCSCs

In accordance with previous reports 4, 27, we employed anisomycin (Sigma-Aldrich) at a final concentration of 31.8 µM (IC_50_ value) in all of our assays. As a control, the same volume of DMSO (Sigma-Aldrich) was applied to the cells in the Ctrl group. The concentrations of butylamine sulfoxide amine (BSO, 20 μM) and elesclomol (24 nM) (Sigma-Aldrich) that we used were as reported previously 14.

### MTT assays

According to previously published methods 27, in briefly, we added 10 μl of 3-(4,5-dimethylthiazol-2-yl)-2,5-diphenyltetrazolium-bromide (MTT) solution (Sigma-Aldrich, St. Louis, USA) to each group of cells for incubation at 37°C for 3h. The formula for calculating the cell proliferation inhibition rate (%) was (1- the OD value of the experimental group of cells-the blank/OD value of the control group of cells-the blank)×100%. Data are depicted as means ± SD where applicable, and differences were evaluated using Student's *t* tests, with a probability of *P* < 0.05 considered to be statistically significant.

### Lipid peroxidation (LPO) assay

The level of LPO products (malondialdehyde, MDA) concentration in cells lysates was assessed using a Lipid Peroxidation (MDA) Assay Kit (Abcam, #ab118970) according to the manufacturer's instructions. Briefly 13, 30, MDA in the sample reacted with thiobarbituric acid (TBA) to generate an MDA-TBA adduct, and this adduct was then quantified color imetrically by observing the OD at 532 nm. We employed C11-BODIPY dye (Thermo Fisher Scientific, USA) to detect lipid peroxidation in cells, as oxidation of the polyunsaturated butadienyl portion of the dye resulted in a shift of the fluorescence emission peak from ~590 to ~510 nm.

### Glutathione assay

The relative GSH concentration in cells was assessed using a GSH/GSSG Ratio Detection Assay Kit (Abcam, #ab205811) according to the manufacturer's instructions. Briefly 13, 30, whole cells were diluted at 1:80 for GSH analysis, and we prepared serial dilutions of GSH and GSSG standards. A one-step fluorimetric reaction of samples with respective assay buffer and probes was incubated for 30 min, and the yellow product (5-thio-2-nitrobenzoic acid) was measured spectrophotometrically at 412 nm.

### Adenosine triphosphate assay

The adenosine triphosphate (ATP) assay was performed according to the manufacturer's protocol from the Enhanced ATP Assay Kit (Beyotime, Shanghai, China) 13, 30. Briefly, 200 μL of the sample lysate buffer (Beyotime) was added to 1×10^6^ cells/mL and this mixture was thoroughly combined by pipetting up and down. The mixture was then centrifuged at 12,000g for 5 min at 4°C, the supernatant was collected, and the ATP standard solutions were also generated. The ATP standard solutions were adjusted to the following concentrations: 0.01, 0.03, 0.1, 0.3, 1, 3, and 10 μM; and they were tested simultaneously with the samples. Fresh testing solutions were prepared as required by the kit's protocol. The ATP testing solution (100 μL) was added to each of the testing wells and standard wells, and these were incubated at room temperature for 5 min. We then added 20 μL of the test sample or standard solution to the wells and quickly mixed them. After 5 seconds at room temperature, the relative light unit (RLU) values were measured using a luminometer.

### cDNA microarray analysis

Total RNA from each group of cells was labeled using Agilent's Low RNA Input Fluorescent Linear Amplification kit, and Cy3-dCTP or Cy5-dCTP was incorporated during reverse transcription of 5 μg of total RNA into cDNA. Different fluorescently labeled cDNA probes were mixed in 30 μL of hybridization buffer (3×SSC, 0.2% SDS, 5×Denhardt's solution, and 25% formamide) and applied to the microarray (Capital Bio Human mRNA Microarray V2.0, CapitalBio, Beijing, China), followed by incubation at 42°C for 16 h. After hybridization, the slides were washed with 0.2% SDS/2×SSC at 42°C for 5 min and then washed again with 0.2×SSC at room temperature for 5 min. The fluorescent images of the hybridized microarray were scanned using an Agilent Whole Human Genome 4×44 microarray scanner system (Santa Clara, CA, USA), and images and quantitative data of the gene-expression levels were analyzed with Agilent's Feature Extraction (FE) software, version 9.5^27^.

### Bioinformatics prediction and analysis

Data from a total of 426 ovarian cancer patients (T) and 88 individuals without ovarian cancer (N) from the Gene Expression Profiling Interactive Analysis (GEPIA, http://gepia.cancer-pku.cn/index.html) were included in the study patient cohorts. These data on patient cohorts were then used for gene-expression profile analysis, pathological stage-plot analysis, multiple gene-comparison analysis, and gene-correlation analysis using the GEPIA online tool (Affy id/Gene symbol: FDX1, DLD, DLAT, LIAS,LIPT1, PDHA, PDHB, ATP7B, YY1) 31.The Kaplan-Meier plotter online tool (https://kmplot.com/analysis/) was applied to the analyses and we plotted survival curves from patient cohorts from a total of 1435 ovarian cancer patients (Affy id/Gene symbol: FDX1, DLD, DLAT, LIAS,LIPT1, PDHA, PDHB, ATP7B, YY1; The follow up threshold: 250 months) 32. The differential gene list subsequently underwent Gene Ontology (GO) and pathway-enrichment analyses using the PANTHER (Protein ANalysis THrough Evolutionary Relationships) Classification System (http://www.pantherdb.org/about.jsp) 33, 34, and the STRING online tool (https://cn.string-db.org) was utilized to construct the protein-protein interaction network (PPI)^35^.

The online software ALGGEN PROMO (http://alggen.lsi.upc.es/cgi-bin/promo_v3/promo/promoinit.cgi) and Tomtom (https://meme-suite.org/meme/tools/tomtom) were exploitedto predict and construct the predictive and basic sequence motif analysis of the gene promoter and transcription-factor binding sites 36-38. The online tool exoRBase 2.0 (http://www.exorbase.org/) was then applied to identify ovarian cancer-associated protein expression in peripheral blood exosomes and the prediction of diagnostic markers 39.

### Statistical analysis

Data are depicted as means ± SD where applicable, and differences were evaluated using Student's *t* tests, with a probability of *P* < 0.05 considered to be statistically significant.With respect to the ANOVA and limma options, genes with a |log2FC| cutoff > 1 and q < 0.01 relative to pre-set thresholds were considered to be differently expressed genes (DEGs). We executed log-rank tests for the survival analysis. Each experiment was performed at least three times. The One-way ANOVA method was uesd for the statistical analysis of multiple comparisons problem. We employed GraphPad Prism 7 software for statistical calculations, and *P*< 0.01 was considered to be statistically significant.

## Results

### Anisomycin has the potential to induce cuproptosis of human ovarian cancer stem cells

**Figure 1:**
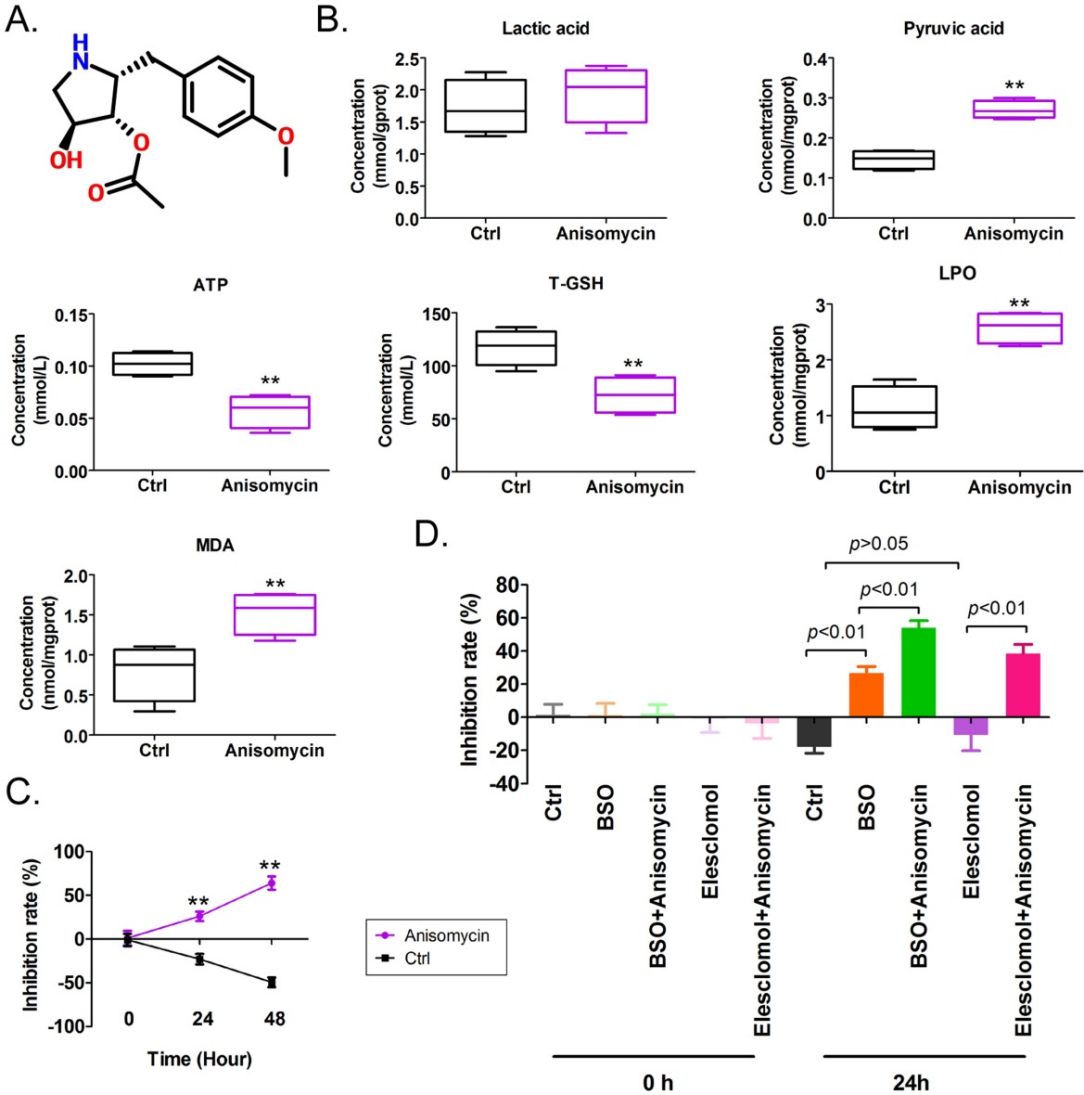
** Anisomycin exhibits the potential to induce cuproptosis in HuOCSCs. (A)**Molecular structure of anisomycin. **(B)**Effects of anisomycin on lipid peroxide, glucose metabolism, and ATP production in HuOCSCs. **P< 0.01 vs. Ctrl group,n=4,*t* test. **(C)**MTT results show that anisomycin inhibited the proliferation of HuOCSCs in vitro.**P <0.01 vs Ctrl group,n=4,*t* test. **(D)**MTT results showed that anisomycin exerted effects similar to those of elesclomol and BSO. One-way ANOVA, n=4.

We found that the IC50 concentration of anisomycin significantly increased intracellular pyruvate content in OCSCs after 24 h, suggesting that anisomycin significantly affected the tricarboxylic acid (TCA) cycle (Figure 1A, 1B). In addition, the ATP and T-GSH contents of anisomycin-treated OCSCs significantly declined, andthe contents of LPO and MDA were significantly elevated relative to control cells (Figure 1B). These results suggested that anisomycin induced the accumulation of lipid peroxides in OCSCs and inhibited cellular detoxification and energy production. MTT results showed that OCSCs treated with an IC50 concentration of anisomycin significantly inhibited cellular proliferation and that the degree of proliferative inhibition was positively correlated with treatment duration (24h: 26.00%±2.68% vs -23.00%±3.03%; 48h: 64.00%±3.76% vs -49.50%±2.78%) (Figure 1C). In addition, the combination of the potent copper-ion carrier elesclomol and the intracellular glutathione-consuming copper-chelating agent BSO along with anisomycin significantly enhanced the inhibitory effect of anisomycin on the proliferation of OCSCs in vitro (BSO+Anisomycin: 54.00%±2.16% vs BSO: 26.75%±1.93%l; Elesclomol+Anisomycin: 38.50%±2.78% vs Elesclomol: -10.75%±4.75%) (Figure 1D). However, the proliferative activity of OCSCs treated with elesclomol alone did not change significantly (Figure 1D). These results showed that anisomycin has the potential to induce cuproptosis of human ovarian cancer stem cells by precipitating lipid-peroxidation damage.

### Anisomycin inhibits the expression of cuproptosis-related genes in human ovarian cancer stem cells

**Figure 2:**
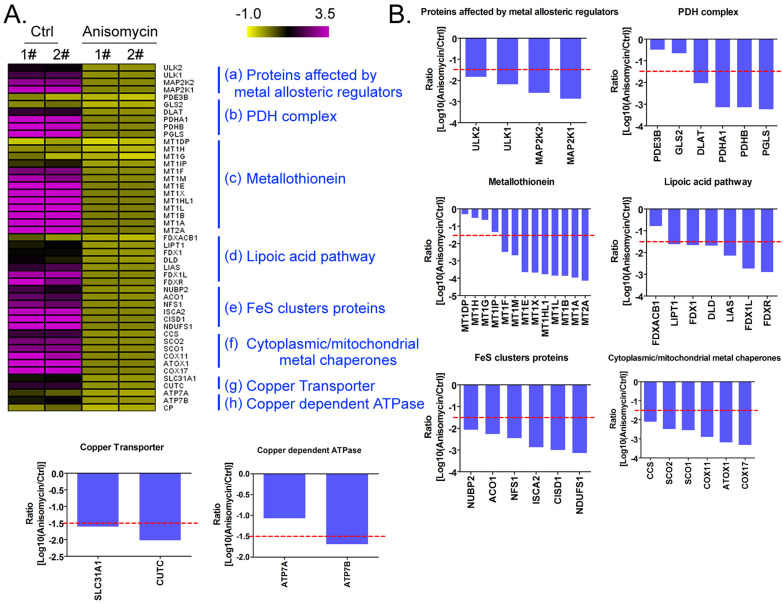
** Anisomycin inhibits the expression of cuproptosis-related genes in HuOCSCs. (A)**Results of thermographic statistical analysis of the transcriptional levels of cuproptosis-related genes using cDNA microarray analysis. **(B)**Anisomycin inhibits the expression of cuproptosis-related genes in HuOCSCs.

**Figure 3:**
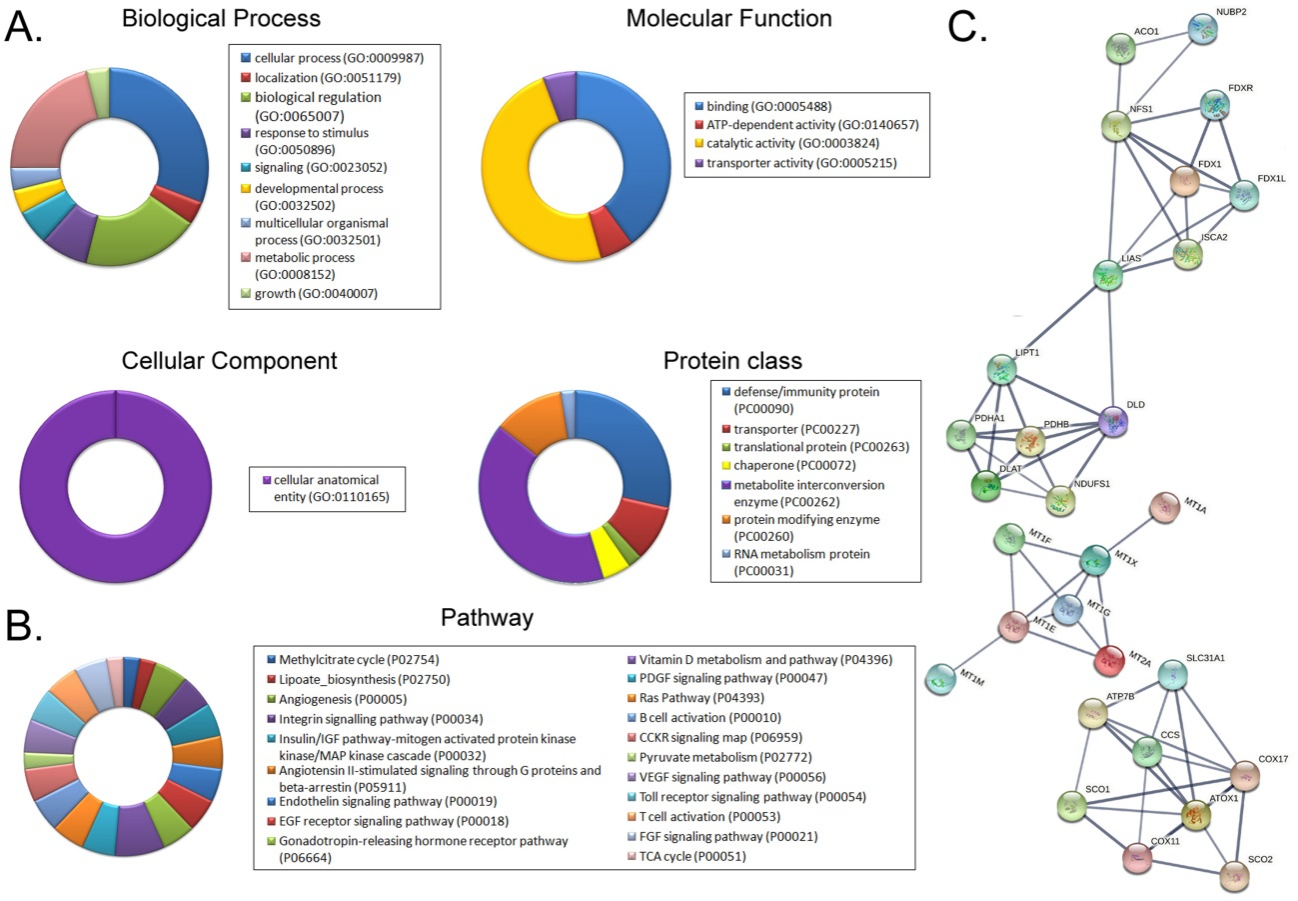
** Functional prediction of differently expressed genes (DEGs). (A)**GO analysis and predictive results. **(B)**Pathway analysis results. **(C)**PPI network prediction results.

Using a cDNA microarray high-throughput detection technology we analyzed the differences in gene expression levels in anisomycin-treated OCSCs group vs DMSO-treated OCSCs (Ctrl) group and noted a total of 28,455 genes, from which we analyzed the expression levels of 47 genes closely related to cuproptosis. These genes were from proteins affected by metal allosteric regulators, PDH complex, metallothionein, lipoic acid pathway, FeS cluster proteins, cytoplasmic/mitochondrial metal chaperones, copper transporter, copper-dependent ATPase, ceruloplasmin, and other categories; and according to previous reports, these genesexert a protective effect on the occurrence of cuproptosis in cells^14^. Our experimental results showed that the expression levels of the aforementioned 47 genes in the anisomycin-treated OCSCs group were significantly attenuated relative to those in the control group (log10 (anisomycin/control) < -1.5; Figures 2A, 2B). These experimental results suggested that anisomycin inhibited the expression of cuproptosis-related genes in OCSCs. The differential gene list subsequently underwent Gene Ontology (GO) and pathway-enrichment analyses using the PANTHER Classification System, and the STRING online tool was utilized to construct the protein-protein interaction network (PPI). GO analysis then indicated that the 47 genes closely related to cuproptosis manifested differential biological effects, including cellular process (Biological process), catalytic activity (Molecular function),cellular morphology entity (Cellular component), and metabolite interconversion enzymes (Protein class) (Figure 3A). Pathway analysis also showed that the 47 genes closely related to cuproptosis were involved in the insulin/IGF pathway-mitogen activated protein kinase kinase/MAP kinase cascade (Figure 3B). PPI network-prediction results ultimately revealed that the proteins encoded by the 47 genes closely related to cuproptosis formed three independent protein-protein interaction networks, involving lipoic acid metabolism pathway, metallothionein, and cytoplasmic/mitochondrial metal chaperones (Figure 3C).

### The expression of lipoic acid pathway components closely related to cuproptosis is closely related to the development and prognosis of ovarian cancer

**Figure 4:**
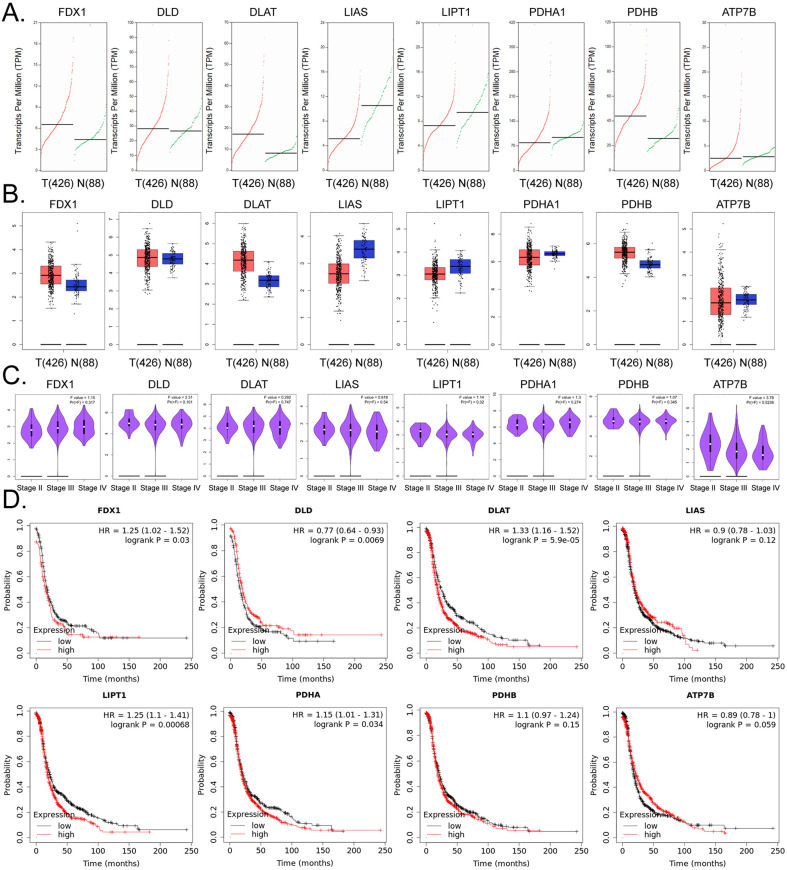
** The lipoic acid pathwayis closely related to the development and prognosis of ovarian cancer. (A)**Transcriptional-level predictive results. **(B)**Results of gene-expression prediction analysis. **(C)**Relationship between the expression of core factors of the lipoic acid pathway and ovarian cancer grade of patients. **(D)**Results of Kaplan-Meier plotter analysis.

Based on the above research results, we further explored the internal relationship between lipoic acid pathway-related factors and the development and prognosis of ovarian cancer. The results of the cDNA expression profile analysis showed that four factors (i.e. FDX1, DLD, DLAT, PDHB) were highly expressed in the tumor tissues of ovarian cancer patients (Figure 4A, 4B). The results of pathological stage-plot statistical analysis depicted expression levels of ATP7B as negatively correlated with the stage of ovarian cancer (Figure 4C), and we noted no significant difference in the expression levels of ATP7B among different stages of ovarian cancer (Figure 4C). Kaplan-Meier plotter analysis showed that except for LIAS, PDHB, and ATP7B, the factors related to PDHB were significantly negatively correlated with the survival cycle of ovarian cancer patients (Figure 4D). These results suggested that the expression levels of core factors in the lipoic acid pathway closely related to cuproptosis were correlated with the development and prognosis of ovarian cancer.

### YY1 is a key transcription factor that regulates the expression of lipoic acid pathway components and affects the malignant degree of ovarian cancer

**Figure 5:**
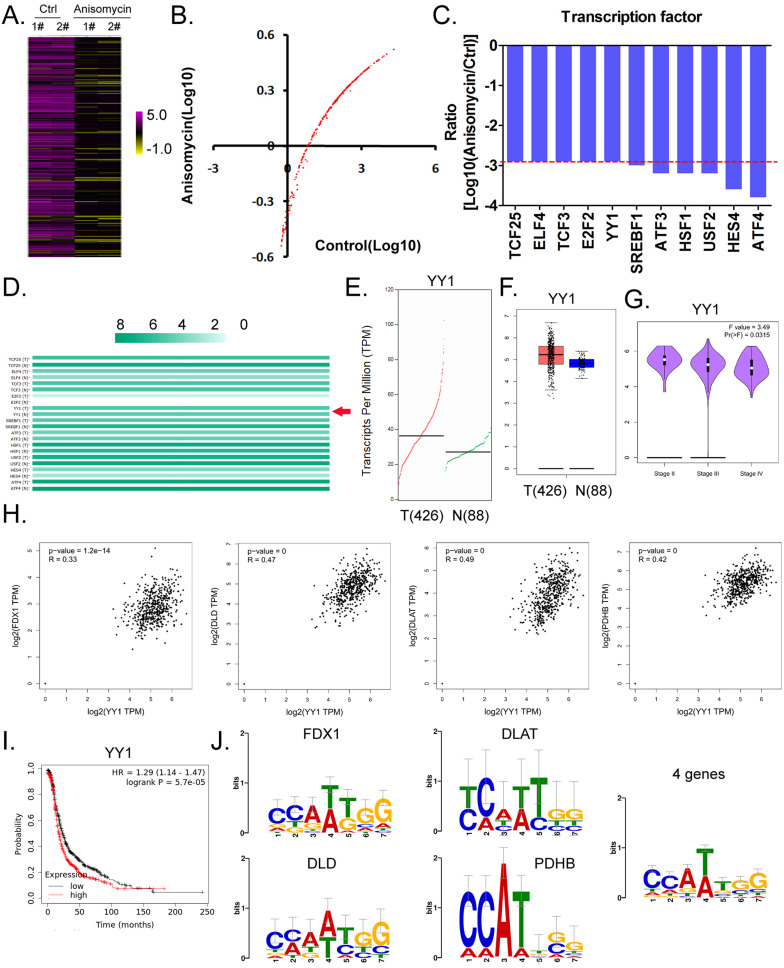
** YY1 is a key transcription factor that regulates the expression of the lipoic acid pathway and affects the malignant degree of ovarian cancer. (A)**Thermographic statistical analysis of transcription-factor expression levels using cDNA microarray analysis. **(B)**Results of scatter-plot analysis of transcription-factor expression levels using cDNA microarray analysis. **(C)**The expression levels of transcription factors were significantly inhibited by anisomycin. **(D)**Analysis results of multiple genecomparisons. **(E)**Results of YY1 transcription-level predictive analysis. **(F)**The predictive results of YY1 expression levels. **(G)** Correlation analysis between YY1 and pathological stage-plot of ovarian cancer patients. **(H)**Correlation analysis between YY1 and core factors of the lipoic acid pathway. **(I)**Results of Kaplan-Meier plotter analysis. **(J)**Results of transcription-factor motif analysis.

Considering the central dogma of molecular biology (DNA→RNA→protein), the first step of gene expression is to activate DNA transcription, and transcriptional activation requires the participation of transcription factors. We analyzed in detail the results related to the expression levels of 186 transcription factors in our cDNA microarray detection data, and found that the expression levels of 11 transcription factors (TCF25, ELF4, TCF3, E2F2, YY1, SREBF1, ATF3, HSF1, USF2, HES4, and ATF4) in the anisomycin-treated OCSCs group were significantly lower than those in the control group (log10 [anisomycin/control]≦ -2.9; Figures 5A, 5B, 5C). The results of YY1 transcription-level predictive analysis was get from the Gene Expression Profiling Interactive Analysis (GEPIA, http://gepia.cancer-pku.cn/index.html). Multiple gene-comparison analysis showed that the expression levels of YY1 were also significantly different between the tumor tissues of ovarian cancer patients and the control group (T vs N: 1.32; *p*<0.05) (Figure 5D). Further analysis showed that the transcription copy number of the YY1 gene in ovarian cancer patients was significantly elevated relative to that in the control group (Figures 5E, 5F).

Pathological stage-plot results revealed that the expression level of YY1 was significantly negatively correlated with the staging of ovarian cancer (Figure 5G); and multiple gene-correlation analysis showed that YY1 gene expressionin ovarian cancer tissues was positively correlated with the expression of FDX1, DLD, DLAT, and PDHB (Figure 5H). The relationship between YY1 expression levels and grade of ovarian cancer patients were analyzed by the Kaplan-Meier plotter online tool (https://kmplot.com/analysis/). The Kaplan-Meier plotter analysis revealed that YY1 was also significantly negatively correlated with the life cycle of ovarian cancer patients (Figure 5I). Finally, transcription-factor motif analysis showed that multiple motif sites were binding to YY1 in the upstream promoter regions of FDX1, DLD, DLAT, and PDHB (length ~ -1000 bp), and that the sites covered the basic motif sequence (C/A)(C/A)(A/G/T)(T/A)(T/G/C)(G/C)] (Figure 5J).

### The levels of YY1 and key lipoic acid pathway factors in peripheral blood exosomes from ovarian cancer patients were significantly higher than those of normal controls

**Figure 6:**
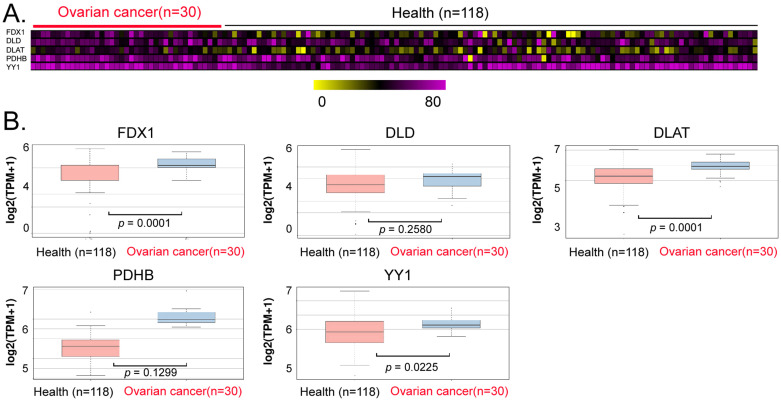
** Exosomal analysis ofperipheral blood from ovarian cancer patients. (A)**Thermographic analysis of the contents of YY1 and key lipoic acid pathway factors in the exosomes from the peripheral blood of patients with ovarian cancer. **(B)**The levels of YY1 and key lipoic acid-pathway factors in the peripheral blood exosomes of ovarian cancer patients were significantly higher than in normal healthy controls.

We explored the potential of YY1, FDX1, DLD, DLAT, and PDHB to serve as non-invasive diagnostic markers of ovarian cancer. We analyzed the differences in the levels of exosomes in peripheral blood between patients with ovarian cancer and normal controls, and our data analysis revealed that the contents of the aforementioned genes (YY1, FDX1, DLAT) in peripheral blood exosomes of the cancer patients were significantly higher (Between 1.2 and 1.5 times) than those in the control group (Figures 6A, 6B). These results suggested that the above factors (YY1, FDX1, DLAT) possessed the potential to be used as non-invasive diagnostic markers for predicting the onset and development of ovarian cancer.

## Discussion

Metal ions have been proven to play a key role in human embryonic development, enzyme activation, and metabolic balance 11-13. Therefore, animbalance inmetal ions may lead to a large number of human diseases, including neurodegenerative diseases and cancer 11-13. Previous studies have shown that copper ionspromote tumor occurrence by facilitating tumor angiogenesis 14-17. Copper, as a basic element of life, enables bacteria, fungi, plants, and animals to thrive; binds to enzymes in the human body; assists inblood coagulatory functions; expedites hormonal maturation; is involved in the cellular processing of energy; and also participates in numerous biological behaviors 14-17. However, excessive copper kills cells and causes pathological damage to multiple organs. Cells normally regulate their copper content via active internal-environment balancing mechanisms and maintain copper at a relatively low level to prevent excessive copper accumulation and cellular damage 14-19. Since initial studies suggested that the intake of copper by a variety of tumor cells was higher than that of normal cells, copper as a research and development target of adjuvant drugs for cancer treatment has gradually attracted increased attention. Guo et al. first reported that Nedd4l-CTR1 regulates AKT kinase in a copper/PDK1-binding manner, and emphasized that AKT-driven destruction of tumor cells can be achieved by targeting the CTR1/copper-signaling pathway 15. Tsvetkov and Golub et al. found that the sensitivity of tumor cells to copper ions depends upon mitochondrial electron-transport-chain energy and was nearly 1000 times higher than that for cells using glycolysis, thus determining that the key gene FDX1 promoted copper-induced cell death. These authors' study also showed that cuproptosis occurred through the direct binding of copper (particularly highly toxic Cu^1+^) to the acylated components of the TCA cycle; this led to lipid-acylation protein aggregation and subsequent loss of iron-sulfur cluster proteins and resulted in protein-toxicity stress that eventually prompted tumor cell death 14. The above research thus indicated the direction for our present research. Our previous studies showed that anisomycin inhibited ovarian cancer stem cell proliferation, invasion, angiogenesis, and tumorigenesis inanimal models 20, 21, 25, 27, 29; our current results also confirmed that anisomycin exerted a multi-targeted regulatory effect 23, 26, 27, 29. But whether anisomycin causes cuproptosis is open to question, and this hypothesis has not been definitively tested thus far. We confirmed that anisomycin combined with the robust copper ionophore elesclomol significantly induced the proliferative inhibition of ovarian cancer stem cells, while elesclomol alone elicited no such effect. Anisomycin combined with the glutathione-depleting copper chelator sulfosulfanyl succinate also significantly facilitated proliferative inhibition of ovarian cancer stem cells. Due to the loss of glutathione and the accumulation of copper ions, the experimental results delineated above indeed suggest that anisomycin carriesthe potential to induce cuproptosis.

We next predicted and analyzed the underlying molecular biological mechanisms and targets of cuproptosis induced by anisomycin in ovarian cancer stem cells via bioinformatics and an open database. In view of previous reports that acylated protein aggregation can lead to protein-toxicity stress and eventually lead to cuproptosis in tumor cells 14, we herein focused on the lipoic acid pathway. Our study revealed that the expression of four core factors of the lipoic acid pathway (i.e., FDX1, DLD, DLAT, and PDHB) was distinct between ovarian cancer patients and normal tissues and that it was significantly correlated with a poor prognosis inovarian cancer patients. This result thus provides a theoretical basis for future treatment. If the induction of cuproptosis is used as adjuvant therapy for ovarian cancer, the target protein needs to be highly expressed and stimulated by corresponding pharmaceutical agents. The lipoic acid pathway, as the core driving signaling pathway of cuproptosis, is crucial with respect to its corresponding copper-ion stimulatory actions. Our theoretical predictive data now directly indicate that there are significant differences in transcripts and expression levels of the core factors of the lipoic acid pathway in tumors and normal tissues; this aspect is novel andcompelling. In addition, the increase atthe gene-transcription level was closely related to transcription-factor activation. We therefore reanalyzed the cDNA microarray analysis data from the ovarian cancer stem cells treated with anisomycin and confirmed that the expression levels of YY1 were significantly reduced in the anisomycin-treated group. Additionally, the results of our bioinformatics analysis showed that YY1 was positively correlated with the expression levels of FDX1, DLD, DLAT, and PDHB. Subsequent promotor motif analysis confirmed that FDX1, DLD, DLAT, and PDHB (and that of other gene promoters) contained multiple binding sites for YY1, and that the basic sequence comprising their binding sites was consistent with the motif reported for YY1. We therefore hypothesize that the direct target of anisomycin-induced cuproptosis in ovarian cancer stem cells is transcription factor YY1. By inhibiting the expression and activity of YY1, anisomycin cannot initiate the transcriptional activity of the core genes in the lipoic acid pathway (i.e., FDX1, DLD, DLAT, and PDHB), and this induces the accumulation of cytotoxic molecules that eventually leads to potential cuproptosis in ovarian cancer stem cells. Indeed the expression levels of 11 transcription factors decreased significantly in the anisomycin treated group according to the results of cDNA microArray. The motif of several transcription factors (i.e., YY1, SREBF1, ATF3, HSF1, USF2, HES4, ATF4, E2F2) on the gene promoters of lipoic acid pathway (i.e., FDX1, DLD, DLAT, and PDHB) have been analyzed by bioinformatics prediction tools. However, the promoter specific sites of above genes contain only the motif of YY1 transcription factor. Therefore, the transcription factor YY1 was as the research object.

In addition, this study extended the mechanism whereby anisomycin activates cuproptosis in ovarian cancer stem cells. We used an exosome database for screening and found that the levels of FDX1, DLD, DLAT, PDHB, and YY1 in peripheral blood exosomes from ovarian cancer patients were significantly higher than those in the normal controls. Furthermore, there has been an increase in studies that entailthe use of exosomal contents in bodily fluids as non-invasive diagnostic markers. Exosomes are formed by intracellular endosomes released into the extracellular environment by exocytosisand range from 20 to 180 nm in diameter. Exosomes contain numerous cellular components, including DNA, RNA, lipids, metabolites, cytoplasm, and surface membrane proteins; and as such, exosomes as carriers of intracellular substances are abundant in peripheral blood and are easily separated and enriched, thereby comprising a potential and sensitive source of noninvasive diagnostic markers.

The experiment results of Figure 1 did not sufficiently show that anisomycin induced cuproptosis as a limitation of this study, because the proliferative inhibition rate was increased when HuOSCSs were cultured with anisomycin and BSO (or elesclomol). The potential effect of anisomycin to induce cuproptosis is the consideration drawn from the total experimental results, including the subsequent gene expression analysis. Meanwhile, the research reports on cuproptosis are very rare, and the reagents (cuproptosis inhibitor or antagonist) that can specifically regulate cuproptosis are also very few. At present, almost no chemical compound has been found to specifically inhibit of cellular cuproptosis. Therefore, it is very difficult to reverse the cuproptosis induced by anisomycin through inhibitor or antagonist of cuproptosis. In future study, we will pay attention to the latest progress in the Field of cuproptosis, and use specific methods to further obtain direct evidence that anisomycin regulates cuproptosis of tumor cells.

In conclusion, our study indicates that the detection of FDX1, DLAT, YY1 levels in the peripheral blood exosomes of patients with ovarian cancer can be used to quickly, accurately, and effectively predict the progress and prognosis of patients with ovarian cancer. Accordingly, the sensitivity and efficacy of cuproptosis-related drug-adjuvant therapy can also be assessed.

## Abbreviations

OCSCs: Ovarian cancer stem cells
BSO: Buthionine sulfoximine
LA pathway: Lipoic acid pathway
Anisomycin: 3,4-Pyrrolidinediol,2-[(4-methoxyphenyl)methyl]-,3-acetate,(2R,3S,4S)
JNK: c-Jun N-terminal kinase
MTT: 3-(4,5-dimethylthiazol-2-yl)-2,5-diphenyltetrazolium-bromide
MDA: Lipid Peroxidation
TBA: Thiobarbituric acid
ATP: Adenosine triphosphate
SOD: Superoxide dismutase
GO: Gene Ontology
PPI: Protein-protein interaction network
DEG: Differently expressed genes
TCA: Tricarboxylic acid
LPO: Lipid peroxidation
